# SOD1 and Amyotrophic Lateral Sclerosis: Mutations and Oligomerization

**DOI:** 10.1371/journal.pone.0001677

**Published:** 2008-02-27

**Authors:** Lucia Banci, Ivano Bertini, Mirela Boca, Stefania Girotto, Manuele Martinelli, Joan Selverstone Valentine, Miguela Vieru

**Affiliations:** 1 Magnetic Resonance Center (CERM), Department of Chemistry, University of Florence, Florence, Italy; 2 FiorGen Foundation, Florence, Italy; 3 Department of Chemistry and Biochemistry, University of California Los Angeles, Los Angeles, California, United States of America; Massachusetts Institute of Technology, United States of America

## Abstract

There are about 100 single point mutations of copper, zinc superoxide dismutase 1 (SOD1) which are reported (http://alsod.iop.kcl.ac.uk/Als/index.aspx) to be related to the familial form (fALS) of amyotrophic lateral sclerosis (ALS). These mutations are spread all over the protein. It is well documented that fALS produces protein aggregates in the motor neurons of fALS patients, which have been found to be associated to mitochondria. We selected eleven SOD1 mutants, most of them reported as pathological, and characterized them investigating their propensity to aggregation using different techniques, from circular dichroism spectra to ThT-binding fluorescence, size-exclusion chromatography and light scattering spectroscopy. We show here that these eleven SOD1 mutants, only when they are in the metal-free form, undergo the same general mechanism of oligomerization as found for the WT metal-free protein. The rates of oligomerization are different but eventually they give rise to the same type of soluble oligomeric species. These oligomers are formed through oxidation of the two free cysteines of SOD1 (6 and 111) and stabilized by hydrogen bonds, between beta strands, thus forming amyloid-like structures. SOD1 enters the mitochondria as demetallated and mitochondria are loci where oxidative stress may easily occur. The soluble oligomeric species, formed by the apo form of both WT SOD1 and its mutants through an oxidative process, might represent the precursor toxic species, whose existence would also suggest a common mechanism for ALS and fALS. The mechanism here proposed for SOD1 mutant oligomerization is absolutely general and it provides a common unique picture for the behaviors of the many SOD1 mutants, of different nature and distributed all over the protein.

## Introduction

Amyotrophic lateral sclerosis (ALS) is a neurological disease that causes the death of motor neurons with consequent muscular paralysis [1]. Although it is predominantly a sporadic disease, 10% of the ALS cases are described as familial (fALS). A link between fALS and mutations in the SOD1 gene was first suggested in 1993 [2], and over 100 fALS-linked mutations, distributed throughout the SOD1 gene, are now associated with approximately 20% of the fALS cases [1], [3], [4]. The pathogenicity of SOD1 mutants has been demonstrated to be due to the gain of a toxic function and not to the loss of the normal function. Thus SOD1 knock-out mice do not show any ALS symptoms, whereas transgenic mice, expressing, for example, the fALS associated mutant G93A human SOD1, develop the symptoms, despite expression of endogenous mouse SOD1 [1], [5]. Studies of the properties of the isolated ALS-mutant SOD1 proteins have *not* revealed the nature of their toxic properties. Some of the mutations differently affect protein stabilities, metal ion affinities and SOD1 activities, while others do not [3], [6], [7], [8]. Thus the molecular mechanisms by which the mutations cause fALS are currently unknown.

Protein aggregates and inclusions are a common pathological feature of many neurological disorders such as Huntington's, Alzheimer's and Parkinson's diseases [9]. In these neurodegenerative diseases, misfolding, aggregation, and precipitation of proteins seem to be directly related to neurotoxicity. The finding of proteinaceous aggregates containing SOD1 in motor neurons of postmortem fALS patients and transgenic mice was therefore a major advance in the field since it suggested that aggregation of SOD1 is related to the pathology of SOD1-linked fALS [10]. As in the other neurodegenerative diseases, it appears unlikely that the visible SOD1-containing inclusions themselves are toxic; rather their presence suggests that smaller, soluble high molecular weight oligomeric precursor species containing SOD1 are being generated *in vivo* [11].

Eukaryotic copper, zinc superoxide dismutase (SOD1) is a 32-kDa homodimeric metalloenzyme, found predominantly in the cytosol, but also in the mitochondrial intermembrane space, nucleus, and peroxisomes. Each of the two subunits of SOD1 forms an eight-stranded Greek key β-barrel and contains an active site that binds a catalytic copper ion (binding residues: His46, His48, His63 and His120) and a structural zinc ion (binding residues: His63, His71, His80 and Asp83). Its functional role is that of catalyzing the dismutation of superoxide radical to dioxygen and hydrogen peroxide [12], [13]. The mature, correctly folded and enzymatically active form of SOD1 is obtained *in vivo* through several post-translational modifications: acquisition of zinc and copper ions, disulfide bond formation, and dimerization [3], [14], [15]. Attention has been focused on how mutations could affect these steps of SOD1 maturation. We have recently shown that wild type (WT) human SOD1, when lacking both its metal ions, forms large, stable, soluble, amyloid-like protein oligomers in solutions exposed to air, under physiological conditions (37°C, pH 7, and 100 µM protein concentration) [16]. Oligomerization was shown to occur through a combination of oxidation of Cys 6 and Cys 111 and formation of amyloid-like interactions between beta strands, as judged by the ability of the oligomers to bind the amyloid-binding dye thioflavin T (ThT), a benzothiazole dye that exhibits increases in fluorescence intensity upon binding to amyloid fibers [17].

The next question therefore was to discover whether fALS-linked mutations in SOD1 oligomerize through the same, common mechanism and, if so, whether this mechanism can be proposed as generally associated to the ALS pathology.

With this aim, we selected a number of mutants, most of which related to fALS. All of them were characterized in the apo and zinc-reconstituted states with respect to their ability to form soluble large molecular weight oligomers. Just as in the WT SOD1, we found that demetallation is the key factor for fALS-mutant SOD1 oligomerization and that intersubunit disulfide bonds involving the free Cys residues, Cys6 and Cys111, as well as formation of ThT-binding non-covalent interactions, uniquely characterize the soluble oligomeric species formed. This sheds a new light on the entire story of ALS and its familial cases. It may be suggested that metal-free SOD1 itself is a cause of ALS and that a number of mutants associated with fALS may be more prone to oligomerization *in vivo*. We found that all of the fALS mutant SOD1 proteins tested, just like WT SOD1, form these high molecular weight oligomers, and that some, but not all, of the fALS mutant SOD1 proteins form them at remarkably fast rates.

## Results

We have recently reported that apo WT SOD1 gives rise to soluble oligomers under aerobic conditions when the protein is kept at 37°C and at a concentration and pH close to physiological, i.e., 100 µM and pH 7. The resulting soluble oligomers are formed by intermolecular disulfide covalent bonds and by non-covalent interactions between beta strands, forming amyloid-like structures capable of binding ThT [16].

**Figure 1:**
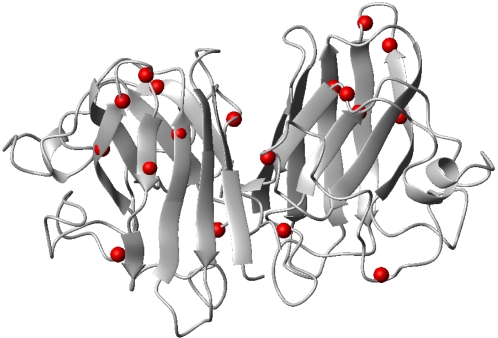
SOD1 investigated mutations. Location of the investigated mutations (red spheres) mapped on the (Cu,Zn) WT SOD1 structure (pdb-ID 1l3n).

The SOD1 mutants (figure 1), which are reported to be linked to fALS disease, were selected following the criteria indicated in Table 1. We selected mutants spread over the entire protein: some of the mutations are located at the subunit-subunit interface, some add a positively charged residue, others substitute an hydrophobic residue with an hydrophilic one, or some produce a simple side chain size variation. Other mutations are on residues either located on secondary structural elements, namely β strands, or just outside them, with change to residues favoring a helical conformation. We also selected a mutation, on a residue in a loop, which introduces a negative charge in the hydrophobic core of the protein. Finally, we introduced two mutations not reported to be related to fALS. Each of these mutant proteins was analyzed with respect to its behavior toward oligomerization, and correlations were sought between the mutant behavior, the nature of the mutation, and its location on the sequence. We purposely introduced the mutations on the “real” WT SOD1 and not on the thermostable form where the two free cysteines (6 and 111) are changed to alanine and serine, respectively (so-called AS SOD1) [18]. This is an essential condition as we have shown that the presence of these two cysteines is the key for SOD1 oligomerization [16].

**Table 1:** Mutants studied in this work and their criteria of selection

Mutations	Criteria of choice
Thr54Arg	Dimer interface, charge change to positive amino acid
Val87Met	Amino acid with α propensity within β strand
Asp90Ala	Amino acid at the protein surface
Gly93Ala	Amino acid with α propensity just outside β strand
Gly93Asp	Charge variation (to negative residue) on buried amino acid
Val97Met	Amino acid with α propensity within β strand
Ile113Phe	Dimer interface, still hydrophobic amino acid
Ile113Thr	Dimer interface, change to hydrophilic amino acid
Leu144Phe	Amino acid with decreased α propensity just outside β strand
Ile35Thr	Non-ALS mutation located on the only SOD1 β strand without mutations; mutation from polar hydrophobic amino acid to non-polar hydrophilic amino acid
Leu67Val	Non-ALS mutation on a pathogenic site located on the zinc-binding loop

Each of the mutants, in the zinc-reconstituted as well as in the apo form, retained the dimeric quaternary structure, as assessed by gel filtration chromatography, which also demonstrated the absence of any significant amount of high molecular weight species. Circular dichroism (CD) spectra on both metallated and apo proteins indicated that the secondary structure present in WT SOD1 was fully conserved in all of the mutants. It has been shown previously that reduction of the intramolecular disulfide bond of apo WT SOD1 causes complete monomerization [15]. We therefore inferred, from the dimeric state of the apo form of all the mutant proteins, that the intrasubunit disulfide bond was intact. For some of the mutants (T54R, V97M and I113T), the folded state of the proteins and the intact disulfide bond were also experimentally confirmed from their ^1^H-^15^N HSQC NMR spectra since their cysteine residues 57 and 146 have shifts very close to those observed for oxidized WT SOD1, but far from those of reduced WT.

Optical and fluorescence spectroscopies, the latter with the use of the ThT, coupled with gel chromatography, showed that the zinc-bound proteins did not give rise to any oligomeric species when they are incubated at pH 7, 37°C, 100 µM concentration, for periods of time longer than a month. The absence of formation of any large molecular weight species was confirmed by gel filtration chromatography. Consistently, turbidity at 400 nm showed no insoluble precipitate. These data indicate that, similar to WT SOD1 [16], the zinc-bound forms of any SOD1 mutant are stable even under prolonged incubation at 37°C.

**Figure 2:**
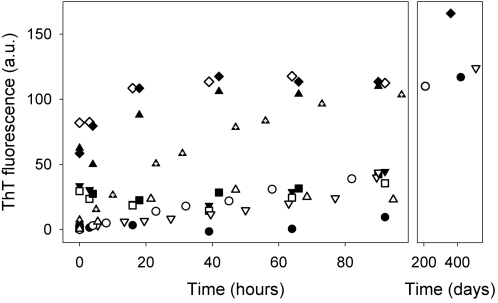
Formation of ThT-binding structures when apo SOD1 mutants and WT are incubated at 37°C. Fluorescence due to ThT binding to SOD1 mutants (presented as arbitrary units, A.U.) for apo T54R SOD1 (•); apo L67V SOD1 (Δ), apo D90A SOD1 (∇), apo I113F SOD1 (□), apo V87M SOD1 (▾), apo WT SOD1 (O), apo L144F SOD1 (▪), apo I35T SOD1 (▴), apo V97M SOD1 (▴), apo G93A SOD1 (◊), apo I113T SOD1 (♦), during the incubation of the samples at 37°C. Apo G93D SOD1 mutant is not reported because the oligomeric species formed precipitates after about 30 hours of incubation.

By contrast, the behavior of the metal-free (apo) form was dramatically different. Upon incubation at 37°C in the air, a progressive increase in ThT-binding fluorescence was observed for the apo form of all the mutants. Figure 2 shows the ThT-binding behavior of the eleven SOD1 mutants, together with that of apo WT SOD1, over a period of more than one year. The temperature dependence of this process was tested for incubations ranging from 15 to 40°C. While at 15°C the oligomerization process is much slower starting after 5–6 days, in the range 36–40°C a difference of one degree Celsius almost doubles the detected rates (data not shown). When a reducing agent such as DTT was added to the solutions of the mutants (tested for T54R, V87M, D90A, G93A, V97M, I113T and L144F), the oligomeric species were destroyed, leading to monomeric species, thus showing that the oligomerization occurs through disulphide bonds. The soluble oligomers, which appear to have a similar amyloid-like structure, as judged by their ThT-binding behavior, are stabilized by H-bond interactions between beta strands of SOD1 subunits. To test further for the existence of these non-covalent interactions, GdnHCl, a cauthropic agent that breaks hydrogen bonds, was added to the oligomers. For each of the mutants tested (T54R, V87M, D90A, I113F, I113T and L144F), the ThT-binding fluorescence was quenched in few minutes, whereas gel filtration of the resulting solutions showed that high-molecular-weight species remained present. While the loss of ThT-binding ability is due to the disruption of the amyloid-like structure of the oligomeric assemblies, the persistence of the oligomeric state is due to the covalent disulfide bonds between the free cysteines of the monomeric subunits.

The rates of oligomerization and consequently of fluorescence increase, is found to depend on the nature of the mutation, being for some mutants strikingly different from that of apoWT SOD1. In particular, three mutants (G93A, V97M and I113T) showed a very fast initial rate of oligomerization, more than twice that of WT SOD1. I35T, a mutant not currently linked to fALS, showed a significantly slower rate of oligomer formation than these three, but the rate was still higher relative to the WT SOD1 protein. The other mutants showed rates of oligomer formation very similar to apo WT SOD1 or, in one case, i.e., T54R SOD1, slightly lower than it. Thus the rates of aggregation for the fALS mutant proteins studied can be divided in two groups, some with rates of oligomerization very similar to that of apo WT SOD1 and three others much faster, with one, non-ALS mutant SOD1 oligomerizing at an intermediate rate. It is important to stress that in no case did a human SOD1 apoprotein fail to form soluble, high molecular weight oligomers. Repeated trials of each of the mutants also established that the kinetics of oligomer formation were highly reproducible as monitored by increases in ThT fluorescence. The data for T54R, D90A, I113T SOD1, and WT SOD1 apoproteins are shown in figure S1.

It is not only the location of the mutation, but also the nature of the amino acid substitution that determines the oligomerization rate. For example, mutation of Ile113 induces a very fast rate of oligomerization, much faster than WT, when is substituted with Thr, but close to that of WT when Ile is replaced by Phe.

Similar oligomerization behavior is also observed for the two mutants, up to now not reported to be involved in ALS. The I35T mutant, located in β3 strand, showed a fast increase in ThT fluorescence (figure 2), and therefore in the rate of oligomerization, as also evidenced by the gel chromatographic analysis (data not shown), while the other mutant here investigated, L67V, ologomerizes with a slower rate, similarly to WT SOD1. The oligomerization of mutants I35T and L67V, in their apo forms, supports our mechanism suggesting that the process would take place for any mutation only when the protein is in the apo form, but not in the zinc-bound form, as we have extensively verified.

In any case, it is important to note that, despite the different aggregation rates, the fluorescence limits at very long times (about 1 year) are similar, indicating the formation of very large molecular weight oligomers for WT and all of the mutant SOD1 proteins studied (figure 2 and figure S1).

**Figure 3:**
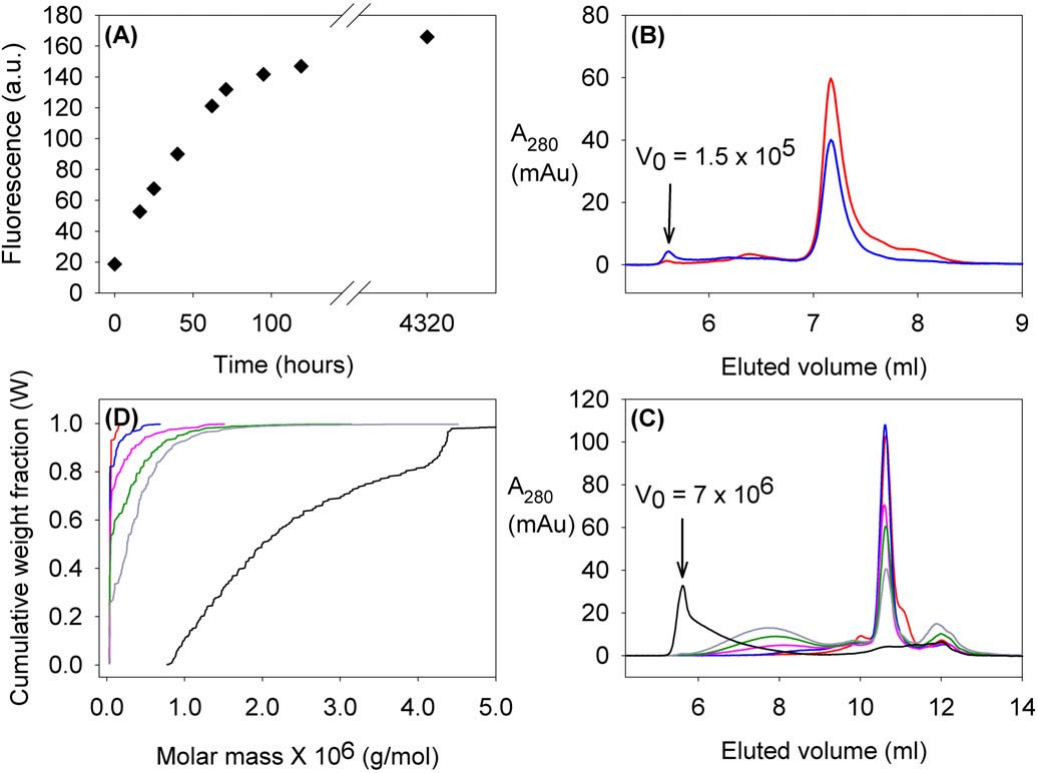
Formation of oligomeric structures when apo I113T SOD1 mutant is incubated at 37°C. (A) Fluorescence due to ThT binding to SOD1 mutants (presented as arbitrary units, A.U.) for apo I113T SOD1 during the incubation of the samples at 37°C. Panels (B) and ( C ) shows the size exclusion chromatograms on a G2000SW_XL_ and a G4000SW_XL _Tosoh columns respectively, corresponding to the samples analyzed by light scattering. The void volume is labeled V_0_. (D) Variation in species distribution during incubation of apo I113T SOD1. Each curve shows the molecular weight distribution detected by light scattering for the sample after different incubation times at 37°C. In all four panels the samples can be identified according to the following colors: before incubation (—), after 16 hours (—), 40 hours (—), 62 hours (—), 4 days (—) and 6 months (—) of incubation at 37°C.

**Figure 4:**
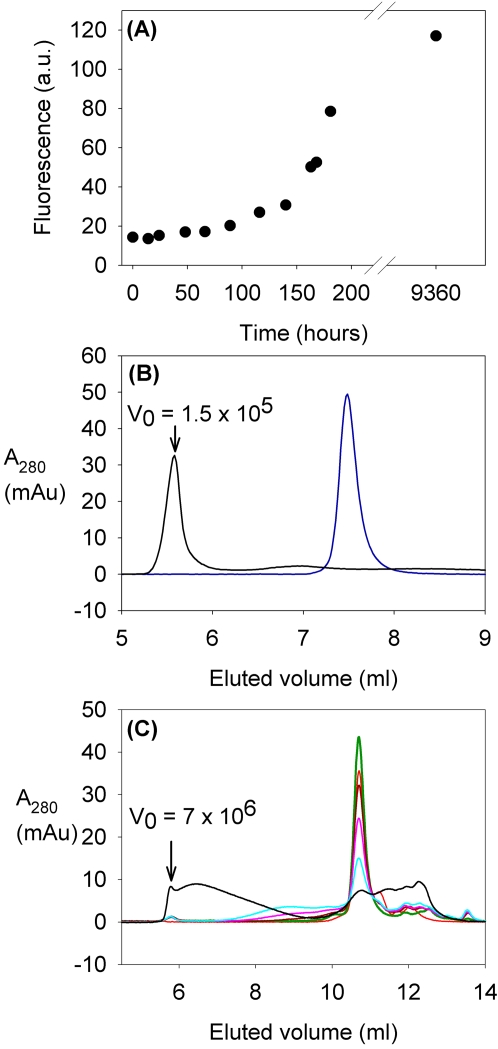
Formation of oligomeric structures when apo T54R SOD1 mutant is incubated at 37°C. (A) Fluorescence due to ThT binding to SOD1 mutants (presented as arbitrary units, A.U.) for apo T54R SOD1 during the incubation of the samples at 37°C. Panels (B) and ( C ) shows the size exclusion chromatograms on a G2000SW_XL_ and a G4000SW_XL _Tosoh columns respectively, corresponding to the samples analyzed by light scattering. The void volume is labeled V_0_. In all four panels the samples can be identified according to the following colors: before incubation (—), after 66 hours (—), 4.8 days (—), 6.8 days (—),7.5 days (—), and 13 months (—) of incubation at 37°C. In panel (B) the zinc reconstituted sample (—) is also reported.

**Figure 5:**
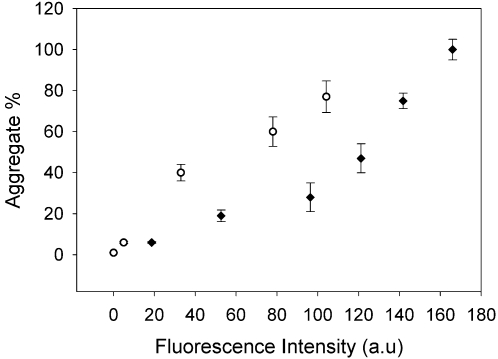
Correlation between percentage of aggregated species and ThT-binding fluorescence. Percentage of aggregated species (non-dimer), determined by light scattering measurements, *vs* ThT-binding fluorescence for apo WT SOD1 (O) and apo I113T SOD1 (♦) during the incubation of the samples at 37°C. Error bars are derived from the molecular mass errors of the light scattering experiments.

The pattern of oligomerization, detected through fluorescence, was paralleled by gel filtration data. The data for the two mutants with the two extreme oligomerization rates, as observed in the fluorescence experiments, (I113T and T54R) are shown in figures 3 and 4. After about 100 hours, while I113T was mostly in high molecular weight states, even if not yet as the final, highest molecular weight ones, mutant T54R was still essentially all in the dimeric state. Gel filtration data also indicated that the final status (after about one year) contains a distribution of high MW species, up to the column cut off (7×10^6^ Da). The presence of intermediate MW species is also observed during the long period of the aggregation process; they eventually evolve towards the final very high MW species. Multi-angle light scattering analyses of the samples along the oligomerization process also showed that the average molecular weight was increasing as a function of time (figure 3, Table S1), with a decrease of the fraction of the dimeric species and the increase of that of the oligomers. The increase in ThT-fluorescence and that in molecular weight from light scattering go in parallel, with a linear correlation between the fraction of aggregated specie (non-dimer) and the ThT-binding fluorescence at each period of incubation (figure 5). This correlation indicates that similar increase in fluorescence corresponds to similar increase in oligomeric species. Multi-angle light scattering data of samples after long periods of incubation, when they reach a steady state condition, provide very similar average molecular weights for all the mutants (of the order of 10^6^ Da), indicating that the oligomers eventually have essentially the same size, independently of the rate of aggregation.

## Discussion

It is now well assessed that, in neuronal tissues of SOD1-linked fALS patients, visible protein aggregates are present that contain aggregated SOD1 and that these aggregates are essentially associated to mitochondria [19], [20]. What is still not understood are the factors and the mechanisms inducing this aggregation. fALS-related SOD1 mutations have been found distributed all over the entire protein and of every possible nature (i.e. charge reversal, charge increase or decrease, from hydrophobic to hydrophilic, different residue size, etc.) with no reasonable correlation/rational between the mutation and the ability to give rise to aggregates. It was already suggested that metal-deficient forms of SOD1 mutants might have a role in fALS [21], [22] but no general relation has been suggested up to now between lack of metal ion and protein aggregation.

Our data on WT SOD1 and on its mutants would then suggest a general feature in the relation between SOD1 and ALS: the apo protein, before it is metalated, is susceptible to oligomerization. Lack of oligomer formation for the metallated proteins shows that metallation is a key factor to inhibit oligomerization. SOD1 or its mutants enter mitochondria as demetallated [23], [15] and, as such, may undergo an abundant oxidation and consequently oligomerization on the way to maturity i.e. before metal binding.

Therefore, independent of the presence and of the nature of the mutation, factors that prevent SOD1 from being efficiently and quickly metalated could lead to protein oligomerization and consequently to disease onset. While it has been shown that the metal-loaded forms of most of the mutant SOD1's are stable and hardly lose the metal ions once metalated as also holds for WT SOD1 [24], several ALS mutations have instead the largest effect on the most immature forms of SOD1; some mutations destabilize the metal-free and disulfide-reduced polypeptide to the point that these forms are unfolded at physiological temperatures [7], [25].

Not all fALS-related mutants enhance the oligomerization process. Actually most of them behave quite similarly to apo WT (figure 2). Only three mutants, out of the eleven investigated, show a significant increase in the initial oligomerization rates compared to the WT protein. We therefore suggest that the presence of a mutation and its location and type would only modulate the rate of the aggregation process. This modulation could occur in two ways: 1) the mutation could influence the local structural and dynamical properties of the apo state, exposing areas of the protein that make it prone to oligomerization through disulfide bond formation between the free cysteines of different SOD1 molecules [16]. The lack of metal ions leads, indeed, to a more disordered SOD1 structure, thus making the free cysteines more exposed, solvent accessible, and less structurally constrained than in the metallated form, as observed in the solution structure of the apo form of a stable monomeric species, characterized by a dramatic increase in protein flexibility, with regions experiencing random coil structural features. This acquired freedom could be affected differently by the various mutations. 2) the mutations could modulate the rate of metalation. If the protein remained for an abnormally long period of time in the apo state, our results suggest that the SOD1 apoprotein, WT or mutant, would be more prone to aggregation.

The aggregation process we observe is strongly sensitive to temperature and obviously to the redox conditions of the environment. Because of the involvement of disulfide bond formation, an oxidative stress would be expected to favor the process, while a reducing environment would prevent aggregation. Indeed, it has been suggested that the redox state of the cell may play a role in the aggregation process [26]. SOD1 is mainly present in two different cell compartments, i.e. cytoplasm and mitochondria, where it independently acquires metal ions. These two cell compartments have quite different redox properties, which could further modulate the aggregation process *in vivo*. It has been proposed that an important toxic property of most mutSOD1s derives from their high level of accumulation in mitochondria in an aggregated state with cross-linked mutSOD1s and that this localization is caused by the aberrant reactivity of cysteines driven by the more oxidizing redox environment of these mitochondria [26]. Recent studies suggest that a non-physiological intermolecular disulfide bond between cysteines at positions 6 and 111 of mutant SOD1 is important for high molecular weight aggregate formation in cells [27].

**Figure 6:**
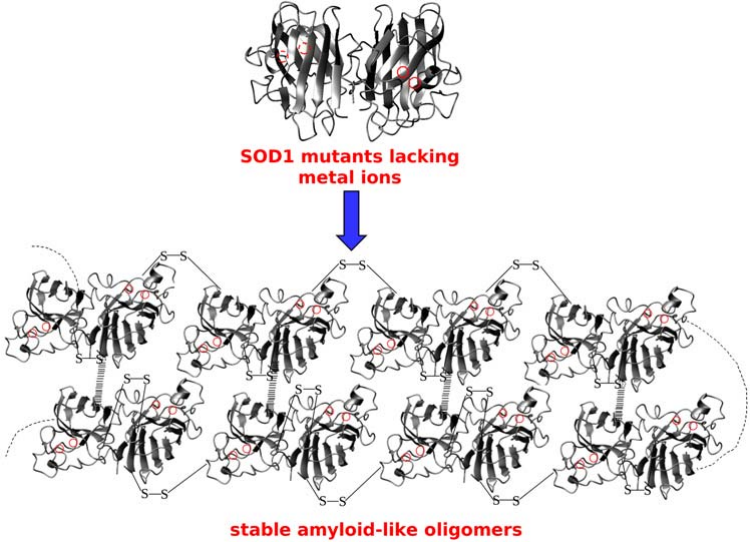
Mutants SOD1 aggregation. Formation of soluble oligomers occuring when apo WT SOD1 protein is kept close to physiological conditions for an extended period of time. In the absence of metal ions, SOD1 proteins form abnormal disulfide cross-links though the two free cysteines (Cys 6 and Cys 111) and noncovalent associations with other SOD1 monomers or dimers.

Our results show that WT, as well as all the SOD1 mutants here studied, form similar high molecular weight oligomers when these proteins, in the apo dimeric form with the intramolecular disulfide bonds intact, are kept in conditions very close to the physiological ones (figure 6). The finding that WT and all the mutants, independently of the nature and location of the mutation, undergo the same type of oligometization suggest a general, unifying picture of SOD1 aggregation that could operate when either wild type or mutant SOD1 proteins are in the metal-free state. Although we cannot exclude other mechanisms in SOD1-linked familial ALS, the one proposed here has the strength of explaining how a large and diverse set of SOD1 mutant proteins all could lead to disease through the same mechanism.

Despite our efforts to build up a correlation between our results and the severity of the disease, the data on the latter are few. The number of cases documented, in particular for the mutations reported by us, is not exhaustive and broad enough to build up a relationship. Still our approach has the potentiality to establish such correlation as soon as enough data become available.

## Materials and Methods

*Sample Preparation—*WT SOD1 and its mutants were expressed in the *Escherichia coli* BL21(DE3) strain. Mutations were performed using a QuikChange™ site-directed mutagenesis kit (Stratagene). The proteins, obtained from cells grown in LB medium, were isolated by osmotic shock in a 20 mM Tris, 5 mM dithiothreitol (DTT) buffer at pH 8. After incubation for 30 minutes at 37°C, the proteins were centrifuged at 40000 rpm for 20 minutes. Supernatants were purified following a reported procedure [28] modified by the addition of 1 mM DTT to each chromatographic buffer. The proteins obtained with this procedure contained substoichiometric amounts of the metal ions (Table S2). The metal ions were completely removed, at 25°C, to prepare the demetallated (apo) form, according to previously published protocols [29], and the zinc reconstituted forms were prepared, as well, as previously described [15]. Metal content of the various forms of SOD1 was checked by inductively coupled plasma mass spectrometry (ICP-MS) using a Thermo Jarrell Ash Atomscan Model 25 Sequential inductively coupled spectrometer (Table S2). The dimeric state of the apo form of the mutants at time zero of the incubation was checked through gel filtration chromatography.

*Spectroscopic characterization—*Protein samples were 100 µM in SOD1 concentration (as dimer) in 20 mM phosphate buffer at pH 7. The proteins were incubated at 37°C to mimic physiological conditions. Optical and fluorescence spectroscopies, coupled with gel filtration chromatography, were used to monitor the formation of oligomeric species at these sample conditions. The analysis were carried out in both the zinc-bound and apo forms of the proteins.

Far-UV CD spectra (190–250 nm) of SOD1 were recorded on JASCO J-810 spectropolarimeter. A cell with a path length of 1 mm was used for the measurement, and the parameters were set as follows: bandwidth, 2 nm; step resolution, 1 nm; scan speed, 20 nm/min; and response time, 2 s. Each spectrum was obtained as the average of four scans. The protein concentration was typically around 8–10 µM. Prior to the calculation of the mean residue molar ellipticity, all of the spectra were corrected by subtracting the contributions from the buffer. Spectra were then smoothed using adjacent averaging or Fast Fourier transform filter. Quantitative estimations of the secondary structure contents were made using the DICROPROT software package [30].

Fluorescence was followed with Thioflavin T, (ThT) probe, which specifically binds to amyloid-like structures [17]. Free ThT has excitation and emission maxima at 350 nm and 450 nm, respectively. However, upon binding to amyloid-like oligomers, the excitation and emission maxima change to 450 and 485 nm, respectively. 54 µl aliquots of sample were added to 646 µl of a 215 µM ThT solution in a 20 mM phosphate buffer at pH 7. The solution fluorescence emission was measured, over time of incubation,with a Cary 50 Eclipse Spectrophotometer supplied with a Single cell Peltier thermostatted cell holder regulated at 37°C. The background fluorescence spectrum of the buffer was subtracted. The excitation wavelength was 446 nm (bandwidth 10 nm) and the emission was recorded at 480 nm (bandwidth 10 nm). Fluorescence intensity at 483 nm was plotted against time of incubation.

Turbidity was measured at 400 nm to detect possible formation of insoluble precipitate. Solution turbidity was measured as apparent absorbance at 400 nm using a Cary UV-visible spectrophotometer. Experiments were performed by diluting 120 µl of the incubation SOD1 stock solution into 280 µl of 20 mM phosphate buffer at pH 7. A 1 cm quartz cuvette was used. Instrumental detection limit was 0.001 at 400 nm.

NMR data were acquired at 288 and 298 K on a 700 or a 900 Bruker spectrometer operating at proton nominal frequencies of 700.13 and 900.13 MHz, respectively. A triple resonance Cryoprobe equipped with pulsed field gradients along the *z*-axis was used. The two-dimensional ^1^H-^15^N HSQC spectra, performed to monitor the folding of the protein mutants, were collected on 100 µM samples of ^15^N-labeled E,E- and E,Zn-hSOD1^SS^ mutant proteins in 20 mM sodium phosphate buffer (pH 7).

*Monitoring SOD1 Aggregation by Gel Filtration and Light Scattering—*100 µl aliquots of the incubated proteins at 37°C were periodically taken and analyzed by gel filtration on Superdex 75 HR 10/30 (Amersham Biosciences) at room temperature. The column was preequilibrated with 20 mM potassium phosphate, pH 7.0, and the flow rate was 0.6 ml/min. The chromatogram, which monitors the species formed during incubation, was obtained by monitoring the absorbance at 280 nm. 20 µl aliquots of the incubated proteins at 37°C were also periodically taken and analyzed by gel filtration on G2000SW_XL_ and G4000SW_XL_ (Tosoh Bioscience) columns at room temperature. The columns were preequilibrated with 20 mM potassium phosphate, pH 7.0, and the flow rates were 0.7 and 1 ml/min respectively. The chromatogram, which monitors the species formed during incubation, was obtained by monitoring the absorbance at 280 nm. A species, present in small amount, was observed in the chromatographic spectra of mutants I113T and T54R, eluting after about 12.0 ml. This species might be due to a fragment from some degradation, which takes place during the aggregation process. We can exclude that it is due to a monomeric species either folded or unfolded as they elute only slightly later than the dimeric species (folded) or even earlier than it (unfolded) (data not shown). Further studies are underway to elucidate this issue.

While Superdex 75 HR 10/30 is a semi-preparative gel filtration columns, the G2000/4000SW_XL_ are analytical ones. Their void volumes are 75 kDa, 150 kDa and 7,000 kDa respectively. The Superdex column was used when a separation of the dimer from the rest of the oligomeric species was necessary for further analysis. The G2000SW_XL_ analytical column was used to monitor the very initial steps of the oligomerization process while the successive time points, in which a larger oligomer, with MW higher than 150 kDa, was formed, were better observed with the G4000SW_XL_ column.

The G2000SW_XL_ and G4000SW_XL_ columns were also connected to a light scattering spectrometer. The online multiangle light scattering (MALS) detector (DAWN EOS, Wyatt Technology, Santa Barbara, CA) and differential refractive index (DRI) detector (Optilab DRI, Wyatt Technology) setup was used to measure the light scattered as a function of angle and absolute protein concentration of fractions eluting from the size-exclusion chromatography column. The Zimm/Debye approximations were used in the Astra software (Wyatt Technology) to estimate molar mass. Data were fit using a second-order polynomial. The analysis was performed for each one of the 20 µl aliquots periodically taken from the incubation batches so as to monitor the increase in molecular weight of the soluble species formed during aggregation.

## Supporting Information

Figure S1 Formation of ThT-binding structures when apo SOD1 mutants and WT are incubated at 37 °C. Fluorescence due to ThT binding to SOD1 mutants (presented as arbitrary units, A.U.) for apo T54R SOD1 (• apo D90A SOD1 (Ñ), apo I113T SOD1 (♦) and apo WT SOD1 (O), during the incubation of the samples at 37 °C. Error bars are standard deviations values obtained from two/three repeats of the experiments.
(0.34 MB TIF)

Table S1 Light Scattering analysis. ThT binding fluorescence, as well as species distribution (dimer and aggregate) and average molecular weights of the aggregated species, as detected by light scattering measurements, of apo I113T SOD1 after different periods of incubation.
(0.02 MB DOC)

Table S2 Extraction from *E. coli* cells and metal reconstitution with zinc. The metal contents of the proteins are shown as equivalents of each metal per enzyme dimer.
(0.02 MB DOC)

## References

[1] Bruijn LI, Miller TM, Cleveland DW (2004). Unraveling the mechanisms involved in motor neuron degeneration in ALS.. Annu Rev Neurosci.

[2] Rosen DR (1993). Mutation in Cu,Zn superoxide dismutase gene are associated with familial amyotrophyc lateral sclerosis.. Nature.

[3] Valentine JS, Doucette PA, Potter SZ (2005). Copper-Zinc Superoxide Dismutase and Amyotrophic Lateral Sclerosis.. Annu Rev Biochem.

[4] Andersen PM (2006). Amyotrophic lateral sclerosis associated with mutations in the CuZn superoxide dismutase gene.. Curr Neurol Neurosci Rep.

[5] Tu PH, Raju P, Robinson KA, Gurney ME, Trojanowski JQ (1996). Transgenic mice carrying a human mutant superoxide dismutase transgene develop neuronal cytoskeletal pathology resembling human amyotrophic lateral sclerosis lesions.. Proc Natl Acad Sci U S A.

[6] Valentine JS, Hart PJ (2003). Misfolded CuZnSOD and amyotrophic lateral sclerosis.. Proc Natl Acad Sci U S A.

[7] Lindberg MJ, Bystrom R, Boknas N, Andersen PM, Oliveberg M (2005). Systematically perturbed folding patterns of amyotrophic lateral sclerosis (ALS)-associated SOD1 mutants.. Proc Natl Acad Sci U S A.

[8] Furukawa Y, O'Halloran TV (2005). Amyotrophic Lateral Sclerosis Mutations Have the Greatest Destabilizing Effect on the Apo- and Reduced Form of SOD1, Leading to Unfolding and Oxidative Aggregation.. J Biol Chem.

[9] Taylor JP, Hardy J, Fischbeck KH (2005). Toxic proteins in neurodegenerative disease.. Science.

[10] Bruijn LI, Houseweart MK, Kato S, Anderson KL, Anderson SD (1998). Aggregation and motor neuron toxicity of an ALS-linked SOD1 mutant independent from wild-type SOD1.. Science.

[11] Ross CA, Poirier MA (2006). Protein Aggregation and Neurodegenerative Disease.. Nat Med.

[12] Fridovich I (1978). The biology of oxygen radicals.. Science.

[13] Bertini I, Mangani S, Viezzoli MS (1998). Advanced Inorganic Chemistry..

[14] Culotta VC, Yang M, O'Halloran TV (2006). Activation of superoxide dismutases: putting the metal to the pedal.. Biochim Biophys Acta.

[15] Arnesano F, Banci L, Bestini I, Martinelli M, Furukawa Y (2004). The unusually stable quaternary structure of human SOD1 is controlled by both metal occupancy and disulfide status.. J Biol Chem.

[16] Banci L, Bertini I, Durazo A, Girotto S, Gralla EB (2007). Metal-free superoxide dismutase forms soluble oligomers under physiological conditions: a possible general mechanism for familial ALS.. Proc Natl Acad Sci U S A.

[17] Krebs MR, Bromley EH, Donald AM (2005). The binding of thioflavin-T to amyloid fibrils: localisation and implications.. J Struct Biol.

[18] Banci L, Bertini I, Cabelli DE, Hallewell RA, Tung JW (1991). A characterization of copper/zinc superoxide dismutase mutants at position 124 - Zinc-deficient proteins.. Eur J Biochem.

[19] Ohi T, Nabeshima K, Kato S, Yazawa S, Tacheki S (2004). Familial amyotrophic lateral sclerosis with His46Arg mutation in Cu/Zn superoxide dismutase presenting characteristic clinical features and Lewy body-like hyaline inclusions.. J Neurol Sci.

[20] Pasinelli P, Belford ME, Lennon N, Bacskai BJ, Hyman BT (2004). Amyotrophic Lateral Sclerosis-associated SOD1 mutant proteins bind and aggregate with Bcl-2 in spinal cord mitochondria.. Neuron.

[21] Stathopulos PB, Rumfeldt JAO, Scholz GA, Irani RA, Frey HE (2003). Cu/Zn superoxide dismutase mutants associated with amyotrophic lateral sclerosis show enhanced formation of aggregates in vitro.. Proc Natl Acad Sci U S A.

[22] Tiwari A, Xu Z, Hayward LJ (2005). Aberrantly increased hydrophobicity shared by mutants of Cu,Zn-superoxide dismutase in familial amyotrophic lateral sclerosis.. J.Biol.Chem..

[23] Field LS, Furukawa Y, O'Halloran TV, Culotta VC (2003). Factors controlling the uptake of yeast copper/zinc superoxide dismutase into mitochondria.. J.Biol.Chem..

[24] Potter SZ, Valentine JS (2003). The perplexing role of copper-zinc superoxide dismutase in amyotrophic lateral sclerosis (Lou Gehrig's disease).. J Biol Inorg Chem..

[25] Lindberg MJ, Tibell L, Oliveberg M (2002). Common denominator of Cu/Zn superoxide dismutase mutants associated with amyotrophic lateral sclerosis: Decreased stability of the apo state.. Proc Natl Acad Sci U. S. A.

[26] Ferri A, Cozzolino M, Crosio C, Nencini M, Casciati A (2006). Familial ALS-superoxide dismutases associate with mitochondria and shift their redox potentials.. Proc Natl Acad Sci U S A.

[27] Niwa J, Yamada S, Ishigaki S, Sone J, Takahashi M (2007). Disulfide Bond Mediates Aggregation, Toxicity, and Ubiquitylation of Familial Amyotrophic Lateral Sclerosis-linked Mutant SOD1.. J Biol Chem.

[28] Banci L, Benedetto M, Bertini I, Del Conte R, Piccioli M (1998). Solution structure of reduced monomeric Q133M2 Copper, Zinc Superoxide Dismutase. Why is SOD a dimeric enzyme?. Biochemistry.

[29] McCord JM, Fridovich I (1969). Superoxide dismutase. Enzymic function for erythrocuprein.. J Biol Chem.

[30] Deleage G, Geourjon C (1993). An interactive graphic programme for calculating the secondary structures content of proteins from circular dichroism spectrum.. Comp Appl Biosc.

