# Supplementary material for: SOD1 and Amyotrophic Lateral Sclerosis: Mutations and Oligomerization
Source: PLoS One. 2008 Feb 27;3(2):e1677. doi: 10.1371/journal.pone.0001677 (PMC2250751; doi:10.1371/journal.pone.0001677)
Supplement: Table S1 — Light Scattering analysis. ThT binding fluorescence, as well as species distribution (dimer and aggregate) and average molecular weights of the aggregated species, as detected by light scattering measurements, of apo I113T SOD1 after different periods of incubation. (0.02 MB DOC) [file pone.0001677.s002.doc]

| Sample  (N) | Incubation  time (hrs) | ThT  Fluorescence | Dimer % | Aggregate % | Aggregate  Average  MM (g/mol) |
| --- | --- | --- | --- | --- | --- |
| 0 | 0 | 18.7 | 94 | 6 | 1.11 × 10^5^ |
| 1 | 16 | 52.7 | 81 | 19 | 1.38 × 10^5^ |
| 2 | 40 | 96.4 | 72 | 28 | 1.98 × 10^5^ |
| 3 | 62 | 121.2 | 53 | 47 | 4.30 × 10^5^ |
| 4 | 95 | 141.8 | 25 | 75 | 4.30 × 10^5^ |
| 5 | ~ 4320 | 166.0 | ~ 0 | ~ 100 | 1.60 × 10^6^ |
