# Supplementary material for: SOD1 and Amyotrophic Lateral Sclerosis: Mutations and Oligomerization
Source: PLoS One. 2008 Feb 27;3(2):e1677. doi: 10.1371/journal.pone.0001677 (PMC2250751; doi:10.1371/journal.pone.0001677)
Supplement: Table S2 — Extraction from E. coli cells and metal reconstitution with zinc. The metal contents of the proteins are shown as equivalents of each metal per enzyme dimer. (0.02 MB DOC) [file pone.0001677.s003.doc]

| Sample | as isolated | | Zinc  reconstituted |
| --- | --- | --- | --- |
|  | Cu | Zn | (E_2_, Zn_2_) |
| WTSOD1 | 0.24 | 0.06 | 1.92 |
| I35T | 0.50 | 0.04 | 1.82 |
| T54R | 0.20 | 0.60 | 1.97 |
| L67V | 0.07 | 0.16 | 1.77 |
| V87M | 0.11 | 0.12 | 1.66 |
| D90A | 0.04 | 0.20 | 1.97 |
| G93A | 0.60 | 0.15 | 1.72 |
| G93D | 0.30 | 0.24 | 1.63 |
| V97M | 0.08 | 0.08 | 1.89 |
| I113F | 0.03 | 0.01 | 1.92 |
| I113T | 0.09 | 0.03 | 2.05 |
| L144F | 0.40 | 0.10 | 1.74 |
